# Uncovering *KRAS*-mutant allele-specific biology and vulnerabilities in non-small cell lung cancer

**DOI:** 10.1042/BCJ20250138

**Published:** 2026-10-27

**Authors:** Paolo D. d'Arienzo, William J. McDaid, Kirsten L. Tinsley, Tong Chen, Emma J. Gasson, Colin R. Lindsay, Angeliki Malliri

**Affiliations:** 1 Division of Cancer Sciences, School of Medical Sciences, Faculty of Biology, Medicine and Health, Wilmslow Road, The University of Manchester, Manchester, U.K.; 2 The Christie NHS Foundation Trust, Manchester, U.K.; 3 Cancer Research UK Lung Centre of Excellence, Manchester, U.K.

**Keywords:** cell signalling, KRAS, non-small cell lung cancer, oncogenes, precision oncology

## Abstract

*KRAS (Kirsten-Ras)* mutations are the most commonly occurring oncogenic drivers in non-small cell lung cancer. Despite the discovery of KRAS inhibitors representing a translational breakthrough for *KRAS*-driven cancers, resistance remains a major clinical challenge. We believe that to circumvent resistance, an in-depth understanding of the biological differences and therapeutic vulnerabilities specific to *KRAS* mutations is critical to inform rational combination partners with either mutant-selective or broad-spectrum (K)RAS inhibitors. The present review draws upon insights from both preclinical and clinical studies exploring *KRAS*-mutant allele-specific behaviours and therapeutic vulnerabilities. We will also highlight studies in which targeting of these vulnerabilities was combined with (K)RAS inhibitors, offering contextual examples of *KRAS*-mutant allele-tailored therapeutic strategies.

## Introduction: KRAS as a tractable driver oncogene in non-small cell lung cancer

Non-small cell lung cancer (NSCLC) is the leading cause of cancer-related deaths worldwide. Based on histopathology, NSCLC is classified into lung squamous cell carcinoma, lung adenocarcinoma (LUAD), large-cell neuroendocrine carcinoma, and other rare subtypes. LUAD is the predominant histological subtype in Western Europe, North America, and Japan, accounting for 50%–60% of total NSCLC cases (Figure 1A) [1–3].

**Figure 1:**
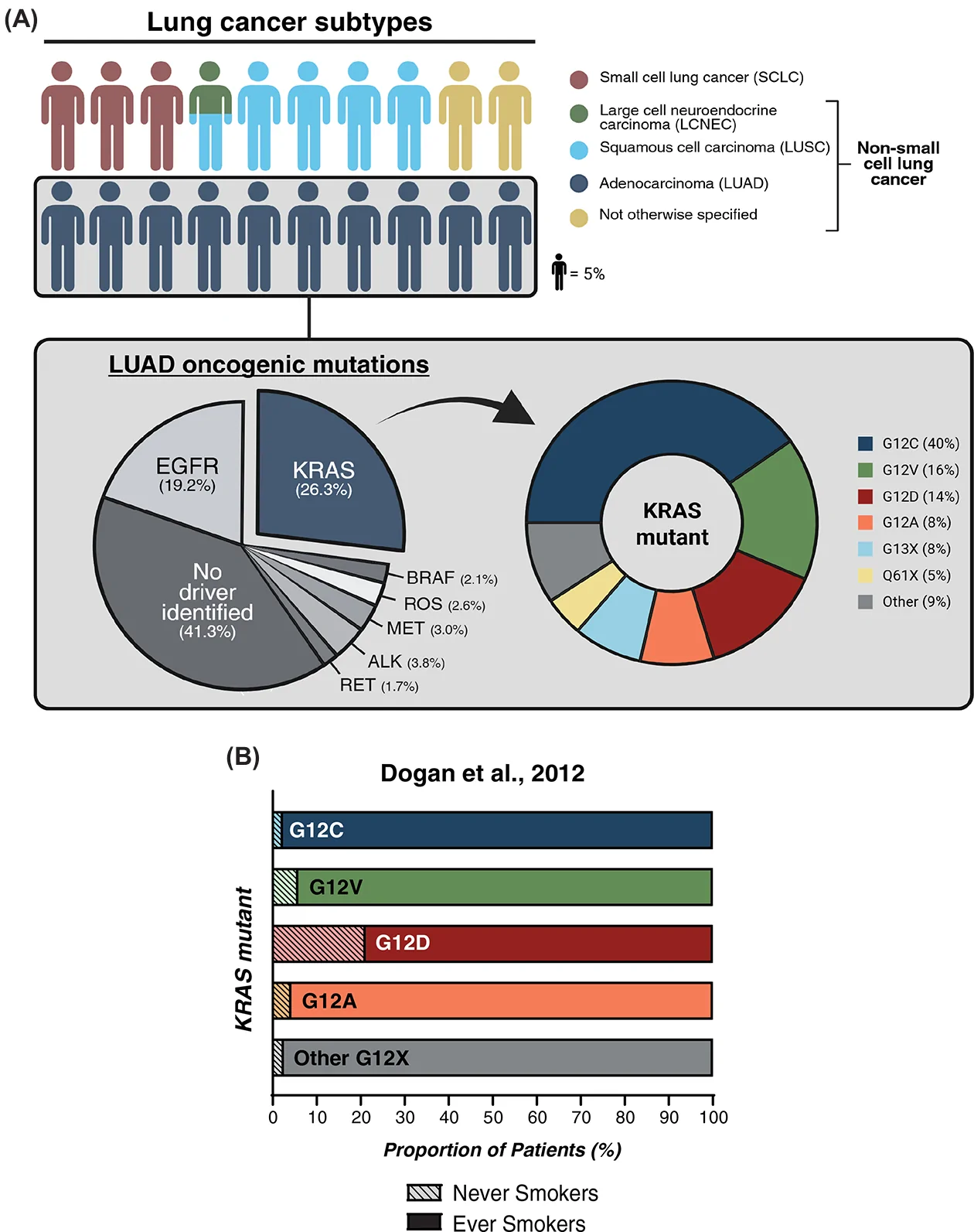
(A) Different *KRAS (Kirsten-Ras)* genotypes in the wider context of non-small cell lung carcinoma and oncogene-addicted LUAD Upper section: distribution of histotypes in NSCLC (data adapted from Figure 1 of Ganti et al., *JAMA Oncol*. 2021) [3]. Lower section: pie charts displaying to the left the frequency of driver genomic alterations in advanced LUAD (data adapted from Figure 1 of Tan and Tan, *J Clin Oncol*. 2022) [4], and to the right the distribution of mutational subtypes in *KRAS*-mutant LUAD (data source: cBioPortal for Cancer Genomics data query, link to studies used in references) [5–7]. Created in BioRender. Tinsley, K. (2027) https://BioRender.com/zaoyfc4. (**B**) Smoking exposure differences across *KRAS* genotypes. Data adapted from Figure 3 of Dogan et al., *Clinical Cancer Res*. 2012 [8]. KRAS: Kirsten rat sarcoma; EGFR: epidermal growth factor receptor; MET: hepatocyte growth factor receptor; ALK: anaplastic lymphoma kinase; RET: rearranged during transfection.

Pathogenic mutations in *KRAS* account for ∼30% of LUAD, making it the most common driver oncogene (Figure 1A) [4,9–11]. Developing effective precision medicine approaches for *KRAS*-mutant cancers has been a formidable undertaking. Nearly four decades elapsed between the identification of *KRAS* as an oncogene in 1984 [12] and the FDA approval of the direct KRAS^G12C^ inhibitor sotorasib in 2021. In comparison, the equivalent process for other targetable NSCLC oncogenes, such as *EGFR* and *ALK*, required 15–25 years. Developing effective KRAS inhibitors has taken longer because of two main factors: (1) KRAS is a small, relatively smooth protein, hindering identification of drug-binding pockets; (2) since KRAS is a guanosine triphosphatase (GTPase) and not a kinase, novel targeted therapy classes had to be invented rather than build upon analogous class precedents [13].

To date, the KRAS^G12C^ inhibitors sotorasib and adagrasib are the only KRAS-targeting drugs to have received regulatory approval for NSCLC, despite extensive preclinical work and ongoing clinical trials. No KRAS-targeting agent has yet demonstrated an overall survival (OS) benefit in NSCLC [14]. In addition to the need for effective targeted therapies for *KRAS*^non^*^-G12C^* mutations, a further major challenge in RAS precision medicine is anticipating treatment resistance. This issue is particularly important, but the breadth of emerging RAS inhibitor combinations being examined would require decades and substantial resources to evaluate comprehensively in clinical trials. Therefore, optimising patient selection and selecting the best combination partners through rational preclinical approaches have the potential to yield substantial clinical benefit by reducing patient exposure to ineffective treatment regimens.

We propose that different oncogene mutations may possess specific properties resulting in unique therapeutic vulnerabilities, a concept that has been proposed before in other oncogene-addicted NSCLC subtypes, such as in *EGFR*-mutant [15,16] and *BRAF*-mutant NSCLC [17,18].

KRAS-mutant proteins differ in biochemical properties, oncogenic properties, and clinicopathological features of disease, indicating that they are ‘not created equal’ [19], and recent studies suggest that they do have distinct therapeutic vulnerabilities [20,21]. We argue that *KRAS*-mutant alleles should be treated as distinct molecular disease subtypes, each with specific systemic therapy (monotherapy or combination therapy) options. In some cases, however, separate mutants may display similar sensitivities to anticancer drugs or drug combinations due to underlying biological similarities and thus may be grouped together for clinical decision-making.

The present review examines the evidence supporting the hypothesis that *KRAS*-mutant NSCLC subtypes function as distinct disease subgroups, with particular emphasis on mutant allele-specific biochemical features, clinicopathologic characteristics, and oncogenicity. From the perspective of informing drug targets and rational combination partners, we will discuss how certain *KRAS* mutations demonstrate unique sensitivity to different classes of KRAS inhibitors and how upstream input and downstream signalling contexts create targetable opportunities. We will primarily focus on reports where at least two *KRAS* mutations were compared.

### An overview of KRAS biology

*KRAS* gain-of-function point mutations are present in approximately 15% of all human tumours [22]. Three genes make up the *RAS* gene family: *KRAS*, *NRAS* (Neuroblastoma-Ras), and *HRAS* (Harvey-Ras), encoding homonymous 21-kilodalton membrane-associated proteins with GTPase enzymatic activity [23]. KRAS exists in two isoforms, KRAS4A and KRAS4B, resulting from alternative splicing. These variants differ only in their carboxyl-terminal residues, with KRAS4B being the predominantly expressed isoform [24].

The RAS proteins are part of a large group of small signal-transduction relay proteins that bind guanine nucleotides and act as molecular switches, alternating between an active GTP-bound state (ON state) and an inactive GDP-bound state (OFF state) [25,26]. When mitogenic stimuli are received, receptor tyrosine kinases (RTKs) are activated and interact with signal transduction proteins located on the inner surface of the plasma membrane, such as the adaptor protein growth factor receptor-bound protein 2 and the tyrosine phosphatase SRC homology-containing protein tyrosine phosphatase 2 (SHP2). Ultimately, activation of signal transduction nodes upstream of KRAS leads to nucleotide exchange (GDP being replaced by GTP), which significantly alters the three-dimensional structure of KRAS: the GTP-bound form contains a ‘loaded-spring mechanism’ in which two hydrogen bonds connect γ-phosphate oxygens to the main chain NH groups of invariant residues in two regions of the G domain known as switch I (residues 32–38) and switch II (residues 59–67), respectively [27]. This conformational change enables KRAS to interact with effector proteins through their specialised RAS-binding domains, mediating several physiological functions associated with cell proliferation, survival, and migration. When the stimulation terminates, KRAS-GTP is converted to its inactive GDP-bound state. Downstream effector pathways include RAF-MEK-ERK (MAPK cascade), PI3K-AKT-mTOR, PLCγ-PKC, TIAM1-RAC, RAL-NF-κB pathways, and others.

Structurally, the KRAS protein comprises two key functional domains: the N-terminal G-domain and the C-terminal hypervariable region (HVR) [28]. The G-domain, responsible for GTP/GDP binding, serves as the core unit for regulating downstream signalling and contains critical structural elements, including the phosphate-binding loop (P-loop; amino acid residues 10–17), switch I (residues 30–38), and switch II (residues 59–67) [29]. The P-loop stabilises the phosphate groups of the nucleotide, whilst the switch regions constitute the interaction interface for guanine nucleotide exchange factors (GEFs), GTPase-activating proteins (GAPs), and downstream effector molecules [28]. The C-terminal HVR exhibits high sequence diversity and contains a CAAX motif (cysteine, two aliphatic amino acids, and a terminal residue). This region undergoes various post-translational modifications (farnesylation, methylation, reversible palmitoylation, and others) that govern the trafficking of the protein to different cellular localisations [30,31]. Consequently, RAS GTPases can interact with different effectors and generate distinct signals. The other main factor underlying differential interactions with effectors is preferential binding between KRAS oncoproteins and certain effectors, which will be discussed later in the present review [31].

The conversion of KRAS to its active GTP-bound form is an intrinsically slow process but is accelerated by GEFs, such as SOS1 and SOS2, which act as catalysts to increase exchange between the GTP- and GDP-bound forms [27]. In a series of fast, reversible reactions, GEFs form a trimeric complex with RAS proteins and GDP to facilitate the ‘driving out’ of the nucleotide and formation of a stable RAS–GEF nucleotide-free complex, followed by rebinding of a guanine nucleotide to RAS; due to the higher concentration of GTP within the cell, this nucleotide is more likely to be GTP than GDP [26].

Similarly, due to the slow intrinsic hydrolytic activity of KRAS, GAPs such as neurofibromin, RASA1, and p120RasGAP are crucial in bringing it back to the inactive GDP-bound state via enhanced GTP hydrolysis [27]. GTP hydrolysis is an irreversible reaction. GAPs do not provide energy for hydrolysis but stabilise the transition state and lower the activation energy required. GAPs bind to RAS-GTP, making the γ-phosphate (the third phosphate attached to guanosine) more accessible to nucleophilic attack and helping in positioning a water molecule near the γ-phosphate, ultimately leading to the conversion of GTP to GDP and inorganic phosphate [26,32].

### KRAS mutational hotspots: biochemical features underlying oncogenic potential

Gain-of-function mutations in *KRAS*, *NRAS,* and *HRAS* in human cancers cluster in three hotspots: codons 12, 13, and 61 [23]. These amino acid residues are conserved, encoding glycine 12, glycine 13, and glutamine 61 in the wild-type (WT) forms of all RAS proteins. All oncogenic amino acid substitutions disrupt the balance between the ON and OFF states, promoting active KRAS and thereby sustaining downstream signalling that drives the acquisition of a malignant phenotype in cells [23].

Substitutions of glycine at codon 12 (*KRAS^G12X^*) are the most common *KRAS* alterations. Amino acid changes at this critical location within the P-loop affect the efficiency with which GAPs can promote KRAS-GTP conversion to KRAS-GDP, introducing an imbalance in favour of the GTP-bound ON state of KRAS. The reason underlying this was identified in early crystallographic studies, which demonstrated that amino acid substitutions at this location cause a steric clash with a catalytic arginine residue provided by GAPs (Arg 1276 in neurofibromin or Arg 789 in p120RasGAP), thereby impeding access to the guanine-binding domain [29,33,34]. The most common mutant in NSCLC, *KRAS^G12C^*, is associated with a mildly reduced intrinsic GTP hydrolysis rate (k_hydrolysis_) versus *KRAS* WT (*KRAS^WT^*), whereas GAP-mediated hydrolysis (using p120GAP) falls by >100 times. In contrast, other *KRAS^G12X^* mutants, such as *KRAS^G12A^, KRAS^G12R^*, and, to a lesser degree, *KRAS^G12D^* and *KRAS^G12V^*, also significantly affect intrinsic KRAS GTPase activity compared with *KRAS^G12C^* [35].

Codon 13 mutations (*KRAS^G13X^*) are the second most common group of *KRAS* mutations in NSCLC. In contrast with Hunter et al. [35], Seamon et al. revealed that several KRAS^G13X^*-*mutant proteins demonstrate comparable intrinsic GTPase activity with KRAS^WT^ [36]. However, both studies showed that this group of mutants increases the proportion of KRAS molecules in the GTP-bound state with a different mechanism: increasing the intrinsic and GEF-mediated rate of GDP-to-GTP exchange (‘hyper-exchange mutants’). Substitutions of the native glycine 13 residue with negatively charged amino acids such as aspartate (*KRAS^G13D^*) or cysteine (*KRAS^G13C^*) destabilise the electro-biochemical status of the nucleotide-binding pocket of KRAS, facilitating the release of bound GDP molecules [37]. This leaves the pocket available, and, due to the higher intracellular concentration of GTP, enhanced nucleotide exchange increases the probability that KRAS binds GTP. Across *KRAS^G13X^* mutations, Hunter et al. examined only the KRAS^G13D^ oncoprotein in their study compared with KRAS^WT^ and a panel of KRAS^G12X^- and KRAS^Q61X^ proteins. Interestingly, KRAS^G13D^ was the only mutant with a significantly different intrinsic GDP exchange rate [35]. Several crystallographic and nuclear magnetic resonance studies have focused on the KRAS^G13D^ mutant and demonstrated its ability to destabilise the nucleotide-binding site of KRAS. This was found to facilitate KRAS-intrinsic nucleotide exchange activity and dramatically increase SOS-mediated nucleotide exchange [35,37–39]. In addition, Rabara et al. reported KRAS^G13D^ and KRAS^G13C^ to be susceptible to neurofibromin-mediated hydrolysis despite being resistant to RASA1 as opposed to KRAS^G12X^-mutant proteins being resistant to both [40]. In contrast with these findings, McFall et al. reported that (1) the affinity of the KRAS^G13D^ oncoprotein for neurofibromin is low and that its GAP activity is less than 1% of KRAS^WT^, and (2) in response to *NF1* transfection in *KRAS^G13D^*/*NF1*-mutant colorectal cancer (CRC) cells, KRAS-GTP levels were essentially unchanged, whereas WT NRAS- and HRAS-GTP were reduced [41–43]. McFall et al. proposed that while KRAS^G12X^ oncoproteins effectively work as competitive inhibitors of neurofibromin, the poor affinity of KRAS^G13D^ for neurofibromin means this can exert its GAP activity on WT HRAS and NRAS in *KRAS^G13D^-*mutant CRC. This could account for the dependence on EGFR signalling on WT RAS protein activation for survival and proliferation. Despite this controversy and the focus being CRC rather than NSCLC, these studies all highlight a significant interplay between KRAS^G13X^ and neurofibromin activity.

Our group, along with Seamon et al., showed that loss-of-function (LOF) mutations in *NF1* occur frequently in *KRAS^G13X^-*mutant NSCLC [36,44]. We showed that *NF1* knockdown increases oncogenic signalling and proliferation in *KRAS^G13D^* NSCLC cells, supporting the hypothesis that *NF1* co-mutations may heighten oncogenic potential in *KRAS^G13X^-*mutant NSCLC [44].

*KRAS^Q61X^* mutations result in amino acid substitutions at residue 61 within the switch II region. Collectively, these variants exhibit reduced GTP hydrolysis and increased nucleotide exchange, representing a ‘hybrid phenotype’ relative to *KRAS^G12X^* and *KRAS^G13X^*. Under physiological conditions, glutamine 61 serves as the catalytic residue that positions and activates the water molecule required for nucleophilic attack on the GTP γ-phosphate. This residue is critical for both intrinsic and GAP-assisted GTP hydrolysis, and its substitution leads to substantial accumulation of KRAS-GTP. In the study by Hunter et al., KRAS^Q61H^ and KRAS^Q61L^ exhibited the most pronounced impairment in GTP hydrolysis, with intrinsic k_hydrolysis_ up to 80 times lower than KRAS^WT^ and p120GAP-stimulated rates up to 1000 times lower than WT [35]. Notably, KRAS^Q61L^ demonstrated an approximately 15-fold increase in k_hydrolysis_ upon GAP stimulation compared with its intrinsic baseline, suggesting that the machinery for GAP-mediated catalysis retains partial functionality in this variant. Gebregiworgis et al. employed a real-time NMR GTPase assay to quantify intrinsic and GAP/GEF-mediated GTP hydrolysis and GDP exchange rates for KRAS^Q61X^ mutants [45]. The intrinsic GTP hydrolysis rate of KRAS^Q61H^ was approximately one-third of that observed with KRAS^WT^; KRAS^Q61H^ was completely insensitive to stimulation by the GAP domain of RASA1, whereas GTP hydrolysis by KRAS^WT^ was increased by more than 300% by RASA1. To evaluate GEF-mediated GDP exchange rates, WT and mutant KRAS proteins were incubated with the recombinant SOS1 catalytic domain, which increased the GDP exchange rate of KRAS^WT^ by more than 20-fold, whereas KRAS^Q61L^ exhibited only a two- to three-fold increase, and KRAS^Q61H^ showed no response [45]. Gebregiworgis et al. presented evidence indicating that glutamine 61 interacts with tyrosine 935 of SOS. While the intrinsic exchange rate of KRAS^Q61H^ is mildly increased as compared with KRAS^WT^, the suppression of SOS binding seen in this mutant prevents the synergistic increases in nucleotide exchange rates observed in KRAS^G13X^ mutants [45].

Summarising all the above, Johnson et al. have proposed a functional classification of oncogenic *KRAS* mutations into four subtypes based on their biochemical features [20]. Class I mutations affect GTP hydrolysis, class II affect nucleotide exchange, class III display a hybrid phenotype, and class IV have no direct effect on the interaction of KRAS with the guanine nucleotide, as they are located away from the active site. *KRAS^G12X^* mutations belong to class I according to this classification, while *KRAS^G13X^* to class II and *KRAS^Q61X^* to class III (Figure 2).

**Figure 2:**
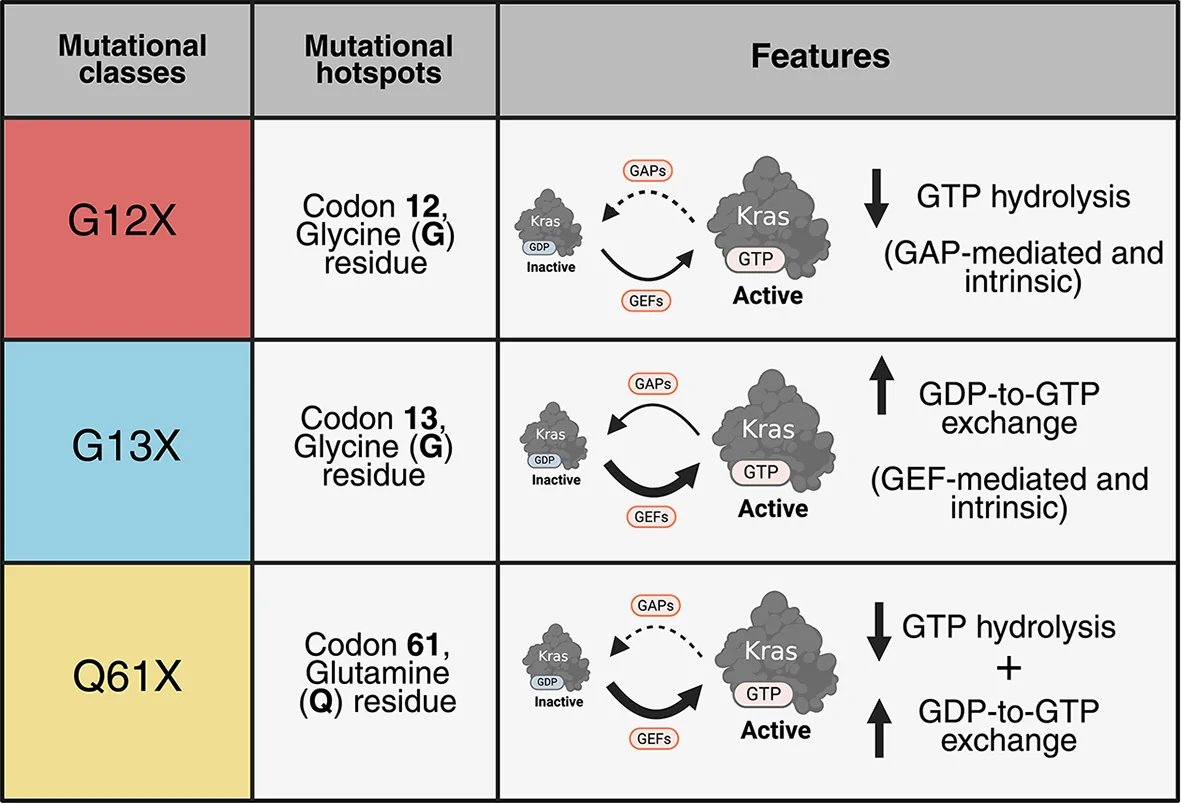
KRAS mutational hotspots: key differences in the GTPase cycle Created in BioRender. Tinsley, K. (2027) https://BioRender.com/fpsdrbu. GAP: GTPase-activating protein; GEF: guanine exchange factor; GDP: guanosine diphosphate; GTP: guanosine triphosphate.

Class IV mutations are rare in human cancers and more commonly seen in germline variants, including non-pathogenic mutations.

### KRAS mutational subtypes in non-small cell lung cancer: clinical and genomic profiles

#### Prevalence

Mutations in the *RAS* proto-oncogenes are among the earliest, if not the first, events in carcinogenesis across several cancers, ranging from carcinomas to non-epithelial cancers such as melanomas and leukaemias [46]. *KRAS* mutations are common in adenocarcinomas, whereas *NRAS* and *HRAS* mutations are more prevalent in melanomas and squamous cell carcinomas, respectively [47]. The tumour type with the highest frequency of *KRAS* mutations is pancreatic adenocarcinoma (90%), followed by adenocarcinomas of the appendix (60%), small bowel (50%), colon/rectum (50%), and NSCLC (30%) [22,23]. Specific cancers are associated with specific amino acid substitutions, suggesting that tissue- and carcinogen-specific factors influence which *KRAS*-mutant subtype confers a fitness advantage [48]. In NSCLC, 80% of *KRAS* mutations are within codon 12, whereas codon 13 is the second most common mutational hotspot, and mutations affecting codon 61 are slightly rarer. Figure 1A includes a graphical representation of the distribution of *KRAS* mutations in LUAD (frequencies derived from cBioPortal for Cancer Genomics query) [5–7].

The glycine-to-cysteine amino acid substitution at codon 12 (henceforth, referred to as *KRAS^G12C^*) is the most common alteration in NSCLC (approximately 40% of all *KRAS*-mutant cases). This is caused by a guanine-to-thymine transversion of the first base of the codon (GGT → TGT; c.34G>T), associated with bulky adducts generated by tobacco smoke mutagens, specifically polycyclic aromatic hydrocarbons. *KRAS^G12V^*, accounting for approximately 20% of *KRAS* mutations, is similarly caused by a guanine-to-thymine transversion, although this affects the second base of the codon (GGT → GTT; c.35G>T). *KRAS^G12D^* is the third most common mutant, making up just under one-fifth of cases, and is instead caused by a guanine to adenine transition in the second base of the codon (GGT → GAT; c.35G>A).

Mutations in codons 13 and 61 make up approximately 5%–7% of all *KRAS* mutants each. Codon 13 mutations are approximately equally divided between *KRAS^G13C^* and *KRAS^G13D^*; *KRAS^Q61H^* is the dominant genotype among codon 61 mutants, making up approximately three-quarters of such cases, whereas *KRAS^Q61L^* represents approximately 20% of all *KRAS^Q61X^* patients.

*KRAS* mutations are prevalent in past or present smokers, although *KRAS^G12D^* has been detected in a higher proportion of never smokers [8] (Figure 1B). A large multicentre study by Ricciuti et al. highlighted the distinctive clinicopathologic profile of *KRAS^G12D^*-mutant NSCLC compared with other *KRAS* mutants. *KRAS^G12D^* cases featured a lower pack-year smoking history overall. Interestingly, smoking exposure in *KRAS^G12D^* follows a bimodal distribution. While 22% of patients with *KRAS^G12D^*-mutant NSCLC were never smokers (versus 4.8% in *KRAS^non-G12D^* NSCLC), 40% still had a smoking history of >30 pack-years [49].

#### Co-mutations

In NSCLC, *KRAS* mutations are frequently accompanied by additional pathogenic genomic alterations in tumour suppressor genes and oncogenes, which influence disease biology and clinical behaviour. Several studies have reported differential co-mutational profiles across *KRAS* mutants.

Arbour et al. described the co-mutation profile in *KRAS^G12C^*-mutant NSCLC compared with *KRAS^non-G12C^-*mutant NSCLC, focusing on *TP53*, *STK11*, and *KEAP1* [50]. *TP53* co-mutations occurred in 43% of *KRAS^G12C^* tumours, *STK11* in 28%, and *KEAP1* in 23%. These proportions were not significantly different from those observed in *KRAS^non-G12C^* tumours.

Judd et al. were the first to provide comprehensive clinicopathologic and genomic information for a large cohort of patients with *KRAS*-mutant adenocarcinoma (4706 patients with KRAS-mutant NSCLC included; *KRAS^G12X^*, 86.5%; *KRAS^G13X^*, 6.9%; *KRAS^Q61X^*, 6.6%), with co-mutation profile data available for all main genotypes [10]. Genes with co-occurring mutations that differed significantly in frequency across genotypes included *STK11*, *KEAP1*, *TP53*, *BRAF*, *NF1*, and *GNAS*. All *KRAS*-mutant subtypes exhibited higher frequencies of *STK11* co-mutations than *KRAS^WT^* tumours, with the highest rate in *KRAS^G13X^* (36.2%) and the lowest in *KRAS^G12D^* (14.2%). *KEAP1* co-mutations were also more common in *KRAS^G13X^*, occurring more than twice as frequently as in other genotypes (13.1% versus 3.7%–6.3%). *TP53* mutations were less frequent in *KRAS*-mutant NSCLC than in *KRAS^WT^* NSCLC; among *KRAS* mutants, the rates ranged between 36% and 52%. *BRAF* mutations were most frequent in *KRAS^G13X^* cases (5.2%) compared with other *KRAS* mutation subtypes (0.7%–2.6%). Most *KRAS* mutants had lower rates of *NF1* co-mutations than *KRAS^WT^* tumours (2.8%–5.1% versus 11.5%), except for *KRAS^G13X^*, which had a notably higher rate (21.4%). *KRAS^G13X^* was also associated with a higher prevalence of high tumour mutational burden than other *KRAS* mutants (50% versus 19%–43%). In summary, the co-mutational landscape varied across *KRAS* mutants, with *STK11*, *KEAP1*, *BRAF*, and *NF1* more commonly observed in *KRAS^G13X^*, and *STK11* considerably less frequent in *KRAS^G12D^*. In *KRAS^Q61X^*-mutant tumours, commonly seen co-mutations include *TP53* (34%–35%), *STK11* (26%), and *KEAP1* (5%–10%) [10].

Despite some heterogeneity across patient cohorts, these patterns were largely replicated by Ricciuti et al. [21], who interrogated the GENIE dataset (*KRAS*-mutant NSCLC: 1131 samples; *KRAS^G12X^*, 88.7%; *KRAS^G13X^*, 6.0%; *KRAS^Q61X^*, 5.8%) to characterise each *KRAS*-mutant co-mutational profile. The high proportion of *NF1* mutations in *KRAS^G13X^* NSCLC across studies is particularly relevant, as LOF mutations in *NF1* may afford additional oncogenic phenotypes to *KRAS^G13X^* NSCLC due to its role as a RAS GAP. In the largest analysis to date focusing on *KRAS^G12V^* (*n* = 636), John and colleagues recently reported very high rates of co-mutations in *STK11* (30.2%) and *KEAP1* (29.3%) [51].

Figure 3 summarises co-mutation frequencies in relevant genes by *KRAS* mutation subtype as determined by querying the cBioPortal for Cancer Genomics [5,6], which aggregated data from multiple published studies, including molecular characterisation of tumours (1636 patients with *KRAS*-mutant NSCLC included) [7]. Once again, similar trends in co-mutation profiles across *KRAS-*mutant alleles were observed: *TP53* and *STK11* co-mutations were the most prevalent across mutational subtypes; *STK11* co-mutations were lower in *KRAS^G12D^* as compared with *KRAS^G12C^*; *STK11*, *KEAP1*, and *NF1* were high in *KRAS^G13X^*.

**Figure 3:**
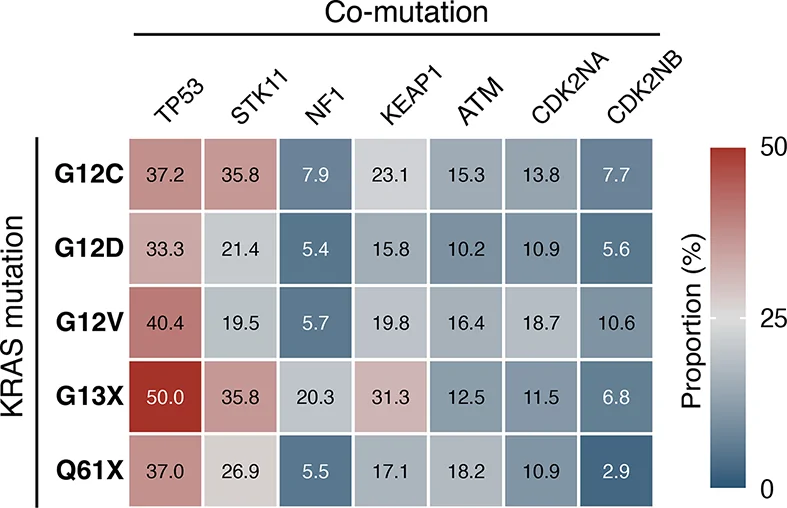
Differential co-mutation profile across *KRAS* mutants in LUAD Source: cBioPortal for Cancer Genomics data query, link to studies used in references [5–7]. Created in BioRender. Tinsley, K. (2027) https://BioRender.com/u9rfwyc.

## KRAS mutation-specific differences in transforming capacity and oncogenic potential

The differences in transforming capacity and oncogenic potential among RAS mutations have been a longstanding question in the field. Early reports focused on *HRAS-* and *NRAS*-mutant variants transduced into mouse or rat fibroblasts, with differences in transforming capacity measured by colony- or foci-formation assays [52,53]. One of the first reports involving KRAS mutations compared *KRAS^WT^*, *KRAS^G12V^*, *KRAS^G12D^*, *KRAS^G13D^*, and *KRAS^Q61H^* in mouse embryonic fibroblasts (MEFs). MEFs expressing any of the four mutant proteins demonstrated foci formation relative to those expressing KRAS^WT^. However, both KRAS^G12X^ mutants showed stronger transforming capacity than the other two mutants [54]. An early explanation for this was that *KRAS^G12X^* mutations were associated with greater activation of the PI3K-AKT-mTOR pathway [55]. With a focus on NSCLC, Ricciuti et al. used an MEF panel where *K-*, *H-*, and *NRAS* were genetically removed, and *KRAS^WT^*, *KRAS^G12C^*, *KRAS^G12D^*, *KRAS^G12V^*, *KRAS^G12A^*, *KRAS^G13D^*, and *KRAS^Q61H^* mutations were transduced, providing a clean system for comparing the impact of these mutations without the confounding effects of WT RAS proteins [21]. They described that all mutations showed strong anchorage-independent growth except for *KRAS^Q61H^*. Furthermore, they inoculated this *KRAS*-mutant MEF panel into mice and assessed *in vivo* growth longitudinally. They reported an *in vivo* survival advantage for *KRAS^G12D^* and *KRAS^G12V^* MEFs, with only *KRAS^G12D^* MEFs forming lung lesions upon histological assessment, concluding that *KRAS^G12D^* had much stronger oncogenic potential.

Other groups have utilised physiologically relevant cell lines and ectopically expressed different *KRAS* mutations to compare their impact on anchorage-independent growth. Ihle et al. used immortalised human bronchial epithelial cells genetically depleted of *TP53* and transduced with *KRAS^WT^*, *KRAS^G12C^*, or *KRAS^G12D^*, observing that expression of KRAS^G12C^ promoted greater anchorage-independent growth over KRAS^G12D^, despite KRAS^G12D^ showing greater PI3K-AKT-mTOR signalling [56].

The use of mouse models with endogenous expression of *KRAS* mutations has provided more elegant comparisons of mutation-driven NSCLC tumourigenesis. As reported by Leone-Kabler et al., the offspring of mice exposed to the carcinogen 3-methylcholanthrene exhibited a spectrum of *KRAS* mutations that depended on the histological stage of the lesion. Of note, *KRAS^G12V^*, *KRAS^G12D^*, and *KRAS^G13R^* were more abundant in LUADs, whereas *KRAS^G12C^* was more abundant in lung hyperplasias, indicating that *KRAS^G12C^* has relatively weaker oncogenic potential *in vivo* despite being the most frequent *KRAS* mutation in NSCLC [57]. In a similar study, Nuzum et al. exposed mice to urethane and saw higher incidences of *KRAS^Q61L^* mutations in lung adenomas and *KRAS^Q61R^* mutations in LUADs, further highlighting differences in oncogenicity between mutations in an *in vivo* setting [58].

Genetically engineered mouse models (GEMMs) provide a more controlled system for comparison of *KRAS* mutation-specific phenotypes. Taking advantage of a well-characterised GEMM of NSCLC that conditionally expresses *KRAS^G12D^* under the control of a Lox-STOP-Lox system [59], our group converted the aspartic acid to a cysteine using CRISPR/Cas9 to create a conditional *KRAS^G12C^*-expressing GEMM, enabling us to directly compare these oncogenes [60]. Using adeno-associated virus (AAV) expressing Cre recombinase to induce expression of each oncogene separately in a time-matched experiment, we saw a striking difference in oncogenic potential, with mice harbouring *KRAS^G12D^* exhibiting multiple large tumours. In contrast, the lungs of *KRAS^G12C^* mice had few small tumours. Additionally, this phenotype was independent of *TP53* loss. We linked the greater oncogenic potential of KRAS^G12D^ with its ability to strongly engage PI3K-AKT-mTOR signalling using isogenic *in vitro* models. In a broader study, Winters et al. used multiplexed AAV/Cas9-mediated homology-directed repair to introduce a barcoded library of specific *KRAS^G12X^* and *KRAS^G13X^* mutations into the lungs of mice [61]. *KRAS^G12X^* mutations led to more tumours than *KRAS^G13X^* mutations, except for *KRAS^G13R^*, which surprisingly exhibited strong oncogenic potential. Consistent with other published studies, among the mutations examined in this study, which are known to occur in NSCLC, *KRAS^G12D^* demonstrated the strongest oncogenicity. Again, this trend was independent of tumour suppressor gene loss, as inactivation of *TP53* or *STK11* did not significantly alter mutation-specific oncogenicity.

Our group also compared codon 12 with codon 13 mutations in NSCLC. We demonstrated that *KRAS^G13X^* mutations confer weaker oncopotency than *KRAS^G12X^* mutations in *in vitro* and *in vivo* models. We linked this phenotype to the inferior ability of *KRAS^G13X^* mutations to activate both the MAPK and PI3K-AKT-mTOR pathways at initiation [44]. We also showed evidence that *KRAS^G13X^*-driven malignant progression requires additional co-mutations, both upstream and downstream of KRAS, in proteins such as neurofibromin, BRAF (non-class I mutations), and LKB1 (*STK11* gene) [44].

Collectively, most studies are consistent in showing intra-codon heterogeneity within codon 12 mutations. *KRAS^G12D^* has strong oncogenic potential, whereas *KRAS^G12C^, KRAS^G12A^*, and most codon 13 mutations are comparatively weaker. These phenotypes appear conserved across cancer types, including PDAC [62,63], suggesting that these *KRAS*-mutant allele-specific differences in oncogenicity are not specific to NSCLC. Although understudied, the data suggest that *KRAS^Q61X^* mutations also display intra-codon heterogeneity. Intriguingly, *KRAS^Q61H^*, despite possessing biochemical features that strongly favour the GTP-bound state, appears to have reduced oncogenicity (in MEFs). This may reflect oncogene-induced toxicity, a hypothesis discussed later in the present review.

## KRAS-mutant allele-specific vulnerability to KRAS targeting

In this section, we will briefly discuss the different generations of KRAS-targeting therapies, with a primary focus on FDA-approved compounds and the landmark discovery of tri-complex ON-state inhibitors. A summary of other KRAS-targeting therapies reported to date is presented in Table 1. We will also describe how the biochemical features unique to certain *KRAS* mutations contribute to their vulnerability to different classes of KRAS inhibitors.

**Table 1:** Clinical trials of KRAS-targeting therapies in NSCLC reported to date

Trial name	Trial phase	Treatment regime	Target	GTPase state targeted	Treatment setting	Size of NSCLC cohort	Objective response rate	Progression- free survival	OS	Rate of grade ≥3 treatment- related adverse events	Data source	Current drug development stage
CodeBreaK 100 [64]	Phase 1–2	RP2D: Sotorasib 960 mg orally once daily	KRAS^G12C^	OFF	Pretreated	174 (pooled phase 1/2)	41%	Median: 6.3 months	Median: 12.5 months; 2-year OS rate: 33%	21%	Peer-reviewed article	Approved by FDA and EMA
CodeBreaK 101 (sotorasib + atezolizumab or pembrolizumab cohorts) [65]	Phase 1–2	Sotorasib + At- ezolizumab 1200 mg or Pembrolizumab 200 mg every 21 days	KRAS^G12C^	OFF	Pretreated	58	29%	Not reported	Median: 15.7 months	Overall: 34/58 (59%); Concurrent: 21/29 (72%); Lead-in: 13/29 (45%)	Conference paper	Approved by FDA and EMA
CodeBreaK 101 (sotorasib + platinum/pemetrexed cohort) [66,67]	Phase 1–2	Sotorasib 960 mg daily + Carboplatin AUC 5 + Pemetrexed 500 mg/m^2^	KRAS^G12C^	OFF	Mixed first-line and pretreated	58 (37 first-line, 21 pretreated)	First-line: 65%; Pretreated: 42%	First-line: median 10.8 months; Pretreated: 8.3 months	Not yet reported	52%	Conference paper	Approved by FDA and EMA
CodeBreaK 200 [68]	Phase 3	Sotorasib 960 mg orally once daily versus Docetaxel 75 mg/m^2^ every 21 days	KRAS^G12C^	OFF	Pretreated	345	Sotorasib: 28% versus Docetaxel: 13%	Sotorasib: 5.6 months versus Docetaxel: 4.5 months; HR: 0.66 (*P =* 0.0017)	Sotorasib: 10.6 months versus Docetaxel: not reported; HR: 1.01 (*P =* 0.53)	Sotorasib: 33% versus Docetaxel: 40%	Peer-reviewed article	Approved by FDA and EMA
SCARLET [68]	Phase 2	Sotorasib 960 mg daily + Carboplatin AUC 5 + Pemetrexed 500 mg/m^2^	KRAS^G12C^	OFF	First-line	30	89%	Median: 6.6 months	Median: 20.6 months	90%	Peer-reviewed article	Approved by FDA and EMA
KRYSTAL-1 [69]	Phase 1–2	RP2D: Adagrasib 600 mg orally twice daily	KRAS^G12C^	OFF	Pretreated	132	43%	Median: 6.9 months	Median: 14.1 months; 1-year OS: 52.8%; 2-year OS: 31.3%	46%	Peer-reviewed article	Approved by FDA and EMA
KRYSTAL-7 [70]	Phase 2	Adagrasib 400 mg orally twice daily + Pembrolizumab 200 mg every 21 days	KRAS^G12C^	OFF	First-line	149	Overall: 44%. PD-L1 <1%: 36%; PD-L1 1–49%: not reported; PD-L1 ≥50%: 59%	Overall, median: 11.0 months; 18-month PFS rate: 37.6%. PD-L1 <1%: 8.2 months; PD-L1 1–49%: 13.5 months; PD-L1 ≥50%: 27.7 months	Overall, median: 18.3 months; 18-month OS rate: 51.8%. PD-L1 <1%: 15.5 months; PD-L1 1–49%: 14.3 months; PD-L1 ≥50%: not reached	64%	Conference paper	Approved by FDA and EMA
KRYSTAL-12 [71]	Phase 3	Adagrasib 600 mg orally twice daily versus Docetaxel 75 mg/m^2^ every 21 days	KRAS^G12C^	OFF	Pretreated	453	Adagrasib: 32%; Docetaxel: 9%	Adagrasib: 6.3 months versus Docetaxel: 3.8 months; HR: 0.58 (*P<*0.0001)	Not yet reported	Adagrasib: 47% versus Docetaxel: 46%	Peer-reviewed article	Approved by FDA and EMA
NCT04449874 (divarasib NSCLC cohort) [72]	Phase 1	RP2D: Divarasib 400 mg orally once daily	KRAS^G12C^	OFF	Pretreated	65	All doses: 53%. RP2D: 59%	All doses: median 13.1 months; RP2D: median 15.3 months	Not yet reported	17%	Peer-reviewed article	Late-phase
LOXO-RAS-20001 (olomorasib monotherapy cohort) [73]	Phase 1	RP2D: Olomorasib 150 mg orally twice daily	KRAS^G12C^	OFF	Pretreated	195 total; 42 KRAS^G12C^ inhibitor-naïve; 41 prior KRAS^G12C^ inhibitor therapy	KRAS^G12C^ inhibitor-naïve: 35%; prior KRAS^G12C^ inhibitor therapy: 41%	KRAS^G12C^ inhibitor-naïve: 7.9 months; prior KRAS^G12C^ inhibitor therapy: 8.1 months	Not yet reported	7%	Peer-reviewed article	Late-phase
LOXO-RAS- 20001 (olomorasib + pembrolizumab cohort) [74]	Phase 1b	Olomorasib 50 or 100 mg orally twice daily + Pembrolizumab 200 mg every 21 days	KRAS^G12C^	OFF	Mixed first-line and pretreated	99 enrolled (51% first-line); 93 treated at 50/100 mg	Overall: 57.1%; first-line: 73.9%; first-line, PD-L1 ≥ 50% subgroup: 90%	Median: 11.8 months (95% CI: 7.6–18.3); 12-month PFS rate: 44.8%	Not yet reported	33.3%	Peer-reviewed article	Late-phase
NCT05009329 (glecirasib monotherapy) [75]	Phase 2b	Glecirasib (800 mg orally once daily)	KRAS^G12C^	OFF	Pretreated	119	47.9%	Median: 8.2 months; 12-month PFS rate: 40.0%	Median: 13.6 months; 12-month OS rate: 54.6%	39%	Peer-reviewed article	Approved by NMPA (China)
NCT05288205 (glecirasib + sitneprotafib) [76]	Phase 1/2a	Glecirasib 400 or 800 mg orally once daily + Sitneprotafib (SHP2 inhibitor) 2 or 3 mg orally once daily	KRAS^G12C^	OFF	Mixed first-line and pretreated	171; 102 first-line; 39 pretreated, KRAS^G12C^ inhibitor-naïve; 30 prior KRAS^G12C^ inhibitor therapy	First-line: 71%; pretreated KRAS^G12C^ inhibitor-naïve: 49%; prior KRAS^G12C^ inhibitor therapy: 10%	First-line: median 12.2 months; ongoing assessment in other subgroups	Not yet reported	46%	Peer-reviewed article	Glecirasib: approved by NMPA (China); sitneprotafib: late-phase
NCT05410145 (elisrasib [D3S-001] NSCLC cohorts) [77]	Phase 1a–1b	Elisrasib (D3S-001) 50–900 mg orally once daily (dose escalation); RP2D: 600 mg orally once daily (dose expansion)	KRAS^G12C^	OFF	Pretreated	22 KRAS^G12C^ inhibitor-naïve; 20 prior KRAS^G12C^ inhibitor therapy	KRAS^G12C^ inhibitor-naïve: 66.7%; prior KRAS^G12C^ inhibitor therapy: 20.0%	Median: not reached in either cohort. KRAS^G12C^ inhibitor-naïve: 6-months PFS rate 66.5%; prior KRAS^G12C^ inhibitor therpay: 6-month PFS rate 44.3%	Not reported	15%	Peer-reviewed article	Late-phase
NCT05533463 (HRS-4642 NSCLC cohort) [78]	Phase 1	HRS-4642 800 mg or 1200 mg intravenously every 2 weeks (dose-expansion)	KRAS^G12D^	ON and OFF	Pretreated	38	23.7%	800 mg every 2 weeks: median 6.3 months; 1200 mg every 2 weeks: median 8.4 months	800 mg every 2 weeks: median 7.5 months; 1200 mg every 2 weeks, median: not reached	24%	Conference paper	Early-phase
RMC-6291-001 (elironrasib NSCLC cohort) [79]	Phase 1	RP2D: Elironrasib 200 mg orally twice daily	KRAS^G12C^	ON	Pretreated	36 KRAS^G12C^ inhibitor-naïve; 24 prior KRAS^G12C^ inhibitor therapy	KRAS^G12C^ inhibitor-naïve: 56%; prior KRAS^G12C^ inhibitor therapy: 42%	KRAS^G12C^ inhibitor-naïve, median: 10.3 months; prior KRAS^G12C^ inhibitor therapy, median: 6.2 months	KRAS^G12C^ inhibitor-naïve, median: not reached, 12-month OS rate 65%; prior KRAS^G12C^ inhibitor therapy, median: not reached, 12-month OS rate: 62%	KRAS^G12C^ inhibitor-naïve: 19%; prior KRAS^G12C^ inhibitor therapy: 8%	Conference paper	Early-phase
RMC-9805-001 (zoldonrasib NSCLC cohort) [80]	Phase 1	RP2D: Zoldonrasib 1200 mg orally once daily	KRAS^G12D^	ON	Pretreated	40 (safety); 27 (efficacy)	52%	Median: 11.1 months; 12-month PFS rate: 48%	Median: not reached; 12-month OS rate: 73%	13%	Conference paper	Early-phase
RMC-6236-001 (daraxonrasib NSCLC cohort) [81]	Phase 1	Daraxonrasib 120–300 mg orally once daily	Multi-RAS- selective	ON	Pretreated	136	≤120 mg subgroup: 31% 160–220 mg subgroup: 34%; 300 mg subgroup: 37%	Platinum and PD-(L)1- pretreated, docetaxel-naive subgroup, median: 8.3 months	Platinum and anti-PD-(L)1- pretreated, docetaxel-naïve, median: 16.0 months	16%	Peer-reviewed article	Late-phase
NCT05382559 (setidegrasib NSCLC cohort) [82]	Phase 1	RP2D: Setidegrasib 600 mg intravenously once weekly	KRAS^G12D^	Not applicable - KRAS degrader	Pretreated	45 (NSCLC at RP2D)	36%	Median: 8.3 months	Median: not reached; 12-month OS rate: 59%	13%	Peer-reviewed article	Late-phase

NSCLC, non-small cell lung cancer; AUC, area under the curve; EMA, European Medicines Agency; FDA, Food and Drug Administration; NMPA, Chinese National Medical Product Administration, OS, overall; PFS, progression-free survival, PD-L1, programmed death receptor ligand 1; RP2D, recommended phase 2 dose; TPS, tumour proportion score.

### The first generation of direct KRAS inhibitors

A critical breakthrough in treating *KRAS^G12C^*-mutant NSCLC was the identification of compounds that covalently bind mutant cysteine 12. Ostrem et al. first identified a drug-binding pocket in the switch II region of KRAS^G12C^ in the OFF state. This led to the discovery of the first molecules that bound to KRAS^G12C^, ‘trapping’ it in its inactive state, switching off KRAS^G12C^ signalling, and reducing proliferation [83]. From here, the first generation of foundational KRAS inhibitors, mostly known as switch II pocket binders, such as ARS853 [84] and ARS-1620 [85], were developed. Building on this, efforts to improve target binding and pharmacodynamic and pharmacokinetic profiles led to the development of the now FDA- and EMA-approved switch II pocket binders sotorasib [86] and adagrasib [87]. These molecules demonstrated anticancer activity in early-phase trials (CodeBreaK 100, KRYSTAL-1) [64,69] in pretreated patients with advanced NSCLC. They also improved progression-free survival compared with standard-of-care docetaxel chemotherapy in pivotal randomised phase III trials (CodeBreaK 200, KRYSTAL-12) [68,71]. However, sotorasib and adagrasib, as monotherapies, produce tumour responses in fewer than half of pretreated patients (29% and 32% in CodeBreaK 200 and KRYSTAL-12, respectively), and, even among responders, acquired resistance often emerges rapidly [88]. Therefore, finding combination partners for KRAS inhibition has been the object of much investigation.

### Next-generation KRAS-targeting therapies

Developing compounds inhibiting other KRAS-mutant proteins has been challenging due to (a) the absence of a cryptic pocket for inhibitors to bind, and (b) the variable reactivities of different amino acid residues.

To date, only a limited number of preclinical studies have reported on non-covalent pan-KRAS inhibitors. BI-2865 and BI-2493 function as pan-KRAS (OFF) inhibitors [89,90], while BBO-11818 and AMG-410 exhibit dual activity against both KRAS (ON) and (OFF) states [91,92]. The argument for directing drug development efforts towards inhibitors with activity against the KRAS (ON) state is that KRAS inhibitors purely targeting the KRAS (OFF) state will have reduced effectiveness (i) against highly activating mutants and (ii) when the tumour microenvironment provides strong upstream stimulation. While AMG-410 has only been described in a conference paper to date [91], a comprehensive report on the activity of BBO-11818 in a number of preclinical models including NSCLC cell lines and cell-derived xenografts has recently been published [92].

A novel strategy for KRAS-targeting drug development is the creation of ‘molecular glue’ drugs. These agents facilitate the interaction between the target and a specific molecule, ‘pasting’ it onto the target and producing protein–protein associations that do not exist under physiological conditions [93]. Molecular glues can affect target function in two ways: (1) tri-complex inhibitors are molecular shields impeding the interaction of the target with downstream effectors and (2) target protein degraders lead to proteasome-mediated proteolysis by facilitating the interaction of the target protein with the E3 ubiquitin ligase complex [93].

Revolution Medicines has developed a series of tri-complex inhibitors that mediate interactions between GTP-bound KRAS and cyclophilin A, an enzyme abundant in the cytoplasm of many cell types. Consequently, a ternary complex (KRAS-GTP/cyclophilin A/inhibitor) is formed, and interaction with effectors is prevented. Peer-reviewed preclinical activity reports are available for mutant-selective inhibitors (*KRAS^G12C^*: elironrasib, previously RMC-6291 [94]; *KRAS^G12D^*: zoldonrasib, previously RMC-9805 [95,80]; *KRAS^G13C^*: RMC-8839 [36]) as well as a non-covalent RAS multi-specific inhibitor (daraxonrasib, previously RMC-6236) [96–99], while others are at an earlier stage of development (*KRAS^G12V^:* RMC-5127 [100]; *KRAS^Q61H^*: RMC-0708 [101]; conference papers only). Tri-complex (K)RAS (ON) inhibitors have demonstrated superior activity compared with KRAS (OFF) state inhibitors, with deeper suppression of MAPK signalling [102]. Results from the phase I cohorts of daraxonrasib and zoldonrasib treatment in pretreated NSCLC have recently been reported. In patients with pretreated RAS-mutant NSCLC, daraxonrasib had response rates of 31%–37% across doses; in the platinum- and anti-PD-(L)1 pretreated, docetaxel naïve subgroup, median progression-free survival (PFS) was 8.3 months and median OS was 16.0 months [81]. The response rate for zoldonrasib in pretreated *KRAS*^G12D^-mutant NSCLC was 52% [80]. These encouraging activity signals have led to trials evaluating daraxonrasib against docetaxel in the pretreated setting (phase III RASolve 301), daraxonrasib in combination with standard-of-care first-line regimens (phase Ib/II NCT06162221 trial, subprotocols A and B), and zoldonrasib monotherapy (phase Ib/II NCT06162221 trial, subprotocol D), which have started recruitment at multiple locations in the U.S.A. and Europe.

Targeting oncogenic KRAS-mediated signalling by promoting KRAS degradation rather than inhibition has been suggested to cause deeper and longer-lasting signalling pathway rewiring. To date, Boehringer Ingelheim has disclosed a pan-KRAS PROTAC (ACBI3; preclinical tool compound only) [103] and Astellas Pharma a KRAS^G12D^-specific PROTAC (setidegrasib, previously ASP3082; in clinical development) [82]. In a phase I trial, setidegrasib had a response rate of 36% (16/45) in patients with pretreated NSCLC who received the recommended phase II dose; median progression-free survival was 8.3 months [82]. Most treatment-related adverse events were grade 1–2 infusion-related reactions, usually during cycle one; grade ≥3 treatment-related event rate was 9%. While advising caution with cross-trial comparison, Park et al. commented that activity appears similar, and the toxicity profile compares positively with the studies reported so far for KRAS^G12D^ inhibitors and for daraxonrasib [82]. ACBI3 is a small-molecule heterobifunctional degrader composed of a non-covalent KRAS switch II pocket ligand chemically linked to a von Hippel-Lindau (VHL) E3 ubiquitin ligase ligand [103]. By comparing head-to-head a pan-KRAS degrader with a stereoisomer that acts as a KRAS inhibitor but cannot bind the VHL E3 ubiquitin ligase, Popow et al. concluded that (a) degraders more potently suppress MAPK signalling, and (b) pharmacodynamic effects last longer than those of inhibitors, extending beyond clearance of the KRAS degrader [103].

The potential therapeutic benefits of a pan-(K)RAS degrader include broad mutation coverage and more durable target suppression. In contrast, reliance on E3 ubiquitin ligase and the proteasome may lead to resistance mechanisms not typical of (K)RAS inhibitors. Comparative efficacy evaluation against (K)RAS inhibitors and assessment of anticancer activity in inhibitor-pretreated patients are open research questions at the time of writing.

Compared with inhibiting each mutant with a specific compound, simultaneous targeting of mutant and WT RAS proteins via pan-(K)RAS inhibitors and degraders has the advantage of being a ‘one size fits all’ strategy and avoiding the development of secondary pathogenic mutations as a tumour escape mechanism. A possible challenge is on-target toxicity due to loss of physiological functions of RAS proteins in normal tissues. However, data reported so far do not necessarily support this. In phase I cohorts of NSCLC patients treated with daraxonrasib, the most frequent treatment-related toxicities were dermatitis, stomatitis, nausea, and diarrhoea; most reported toxicities were mild or moderate in severity [96,104]. Whether sparing the NRAS and HRAS isoforms with a pan-KRAS targeted therapy may lead to lower rates of drug-related toxicity in comparison with daraxonrasib is currently unknown.

### KRAS-mutant allele-specific vulnerability to OFF-state inhibitors

Switch II pocket binders demonstrated that KRAS^G12C^ possessed GTPase activity and still depended on RTK-driven GEF activity to an extent. This was surprising, given that at the time it was still believed that mutant KRAS was fully independent of upstream signals and existed in an ON state. Two seminal publications using the first generation of switch II pocket binders revealed how KRAS^G12C^ relies on RTK activation and GEF modulation. Firstly, Lito et al. showed that this feature contributed to the activity of the switch II pocket binder ARS853. By generating a KRAS^G12C^ protein with abolished GTPase activity, they reported that the compound had diminished activity [105]. They further highlighted that RTK inhibition can modulate nucleotide exchange, enhancing switch II pocket binder efficacy. Using heterozygous and homozygous *KRAS^G12C^* models (the latter of which removes the potential confounding impact of KRAS^WT^), they showed that inhibiting different RTKs, such as EGFR and FGFR, improved the impact of ARS853 by expanding the pool of OFF-state KRAS^G12C^. Secondly, a complementary study by Patricelli et al. demonstrated that the cellular engagement of ARS853 with KRAS^G12C^ and subsequent downstream pathway inhibition correlated with a nucleotide exchange kinetic model, supporting the conclusion that KRAS^G12C^ was modulated by GEF activity [84]. They further supported the work by Lito et al. and demonstrated that epidermal growth factor (EGF) stimulation reduced ARS853 activity by promoting KRAS^G12C^-GTP, and, conversely, that EGFR inhibition potentiated ARS853 activity by expanding the pool of GDP-bound KRAS^G12C^.

Another series of OFF-state inhibitors targeting pan-KRAS, known as BI-2493 and BI-2865, confers broad activity in *KRAS*-mutant cancer cell lines (such as those expressing KRAS^G13D^, KRAS^G12D^, or KRAS^A146T^), including higher activity in *KRAS^WT^*-amplified tumours, a type of *KRAS* alteration not frequently seen in NSCLC [106]. As KRAS^WT^ has intact GTPase functioning, these compounds also rely on this feature to exert their anti-tumour effects. The contribution of high GTPase activity and nucleotide exchange capability towards the activity of OFF-state KRAS targeting raises the question of which other *KRAS* mutations beyond *KRAS^G12C^* are vulnerable to this type of therapeutic intervention. Informed by the reports from Hunter et al. [35] and Seamon et al. [36], which both investigated the differences in nucleotide exchange and GTPase activity between KRAS mutants, it seems likely that only a small handful of mutations would ultimately benefit from this form of targeting: for example, *KRAS^G12C^*, *KRAS^G12D^*, and *KRAS^G13D^*. Mutation-specific inhibitors targeting the OFF state of KRAS^G12D^ exist (e.g., MRTX1133) [87]. However, they do not exist for KRAS^G13D^. It is plausible that other codon 13 mutants may behave similarly to KRAS^G13D^ and respond well to mutation-specific OFF state inhibition. Using a large isogenic *KRAS*-mutant HEK293 panel expressing several KRAS^G12X^, KRAS^G13X^, KRAS^Q61X^, and other rare mutants, Kim et al. demonstrated increased sensitivity of KRAS^G13C^ and KRAS^G13D^ to OFF-state pan-KRAS inhibition, as evidenced by complete dose-dependent suppression of KRAS-GTP [89]. Several mutants, such as KRAS^G12V^, KRAS^G12R^, KRAS^Q61H^, and KRAS^Q61L^, which are predominantly in the ON state, did not show complete suppression of KRAS-GTP, once again supporting the hypothesis that, in general terms, developing inhibitors capable of targeting the ON state is preferable.

### KRAS-mutant allele-specific vulnerability to ON-state inhibitors

The discovery of tri-complex inhibitors targeting the ON state of RAS helped bypass the complex resistance mechanisms mounted in response to OFF-state inhibitors and uncovered novel differences among *KRAS* mutations in their susceptibilities to ON-state targeting [99]. Using the multi-selective RAS (ON) inhibitor RMC-7977, Holderfield et al. conducted a large drug–response screen involving cell lines harbouring several types of RAS pathway alterations and revealed a correlation between *KRAS* mutational subtype and compound activity [99]. *KRAS^G12X^*-mutant cell lines were more vulnerable to this RAS-targeting modality, with a 10-fold difference in EC_50_ between *KRAS^G12X^* and *KRAS^non-G12X^*-mutant cell lines. This correlated with *KRAS* gene dependency data, also showing that *KRAS^G12X^*-mutant cell lines depended more on KRAS for survival. They reasoned that the difference in response to RMC-7977 between *KRAS^G12X^* and *KRAS^non-G12X^*-mutant cell lines is due to the distinct biochemical features of these mutations, previously described in the present review. Jiang et al. conducted a similar investigation using another multi-selective RAS (ON) inhibitor, RMC-6236, and observed activity against both KRAS^G12X^ and NRAS^Q61X^ mutants [97]. Within the *KRAS^G12X^*-mutant subset of cell lines, KRAS^G12V^ mutants were overall most sensitive to RMC-6236, which is plausible, given that it has lower GTPase activity than KRAS^G12C^ and KRAS^G12D^ in the report by Hunter et al. [35].

The relative resistance of *KRAS^G13X^*-mutant cell lines to multi-selective RAS (ON) targeting casts doubt on targeting this genetic subset of cancer through this type of intervention. Given their biochemical features, they may be more effectively targeted with an OFF-state inhibitor. However, no KRAS^G13X^-selective OFF-state inhibitors had been reported until a recent study by Seamon et al., where a mutation-tailored combination strategy involving a multi-selective RAS (ON) inhibitor and a mutant-selective inhibitor to target *KRAS^G13C^*-driven NSCLC was used [36]. Interestingly, the authors observed greater variability in the depth of pERK1/2 suppression in *KRAS^G13C^*-driven cancer cell lines treated with a mutant-selective inhibitor (RMC-8839) compared with *KRAS^G12X^* cell lines, which all showed complete inactivation of pERK1/2 in response to their respective selective ON-state inhibitors. They noted that the residual pERK1/2 activation in *KRAS^G13C^* cell lines was due to WT RAS activation. Their analyses of patient data also showed higher incidences of *NF1* mutations and RTK mutations in *KRAS^G13X^* patients. Their data overall suggested that WT RAS activity, bolstered by *NF1* LOF mutations and increased RTK signalling, which increases WT RAS-GTP, may play an important role in *KRAS^G13X^*-mutant tumours (consistent with the retrospective analysis of cetuximab activity in *KRAS^G13D^*-mutant CRC [107,108]) and may have contributed to reduced activity of RMC-8839 in *KRAS^G13C^*-driven models. They used the RAS (ON) multi-selective inhibitor RMC-7977 to block RTK-driven activation of WT RAS proteins, in combination with RMC-8839, to disable KRAS^G13C^, thereby improving anti-tumour activity.

Holderfield et al. and Jiang et al. also reported reduced activity of multi-selective RAS targeting in *KRAS^Q61X^*-driven cancers [97,99]. This was consistent with their *KRAS* dependency data, which showed that *KRAS^Q61X^*-driven cell lines are less dependent on KRAS for survival. This adds another layer of complexity to this subset of *KRAS* mutations, given that they exhibit low GTPase activity, like that of *KRAS^G12V^* [35]. Although no mechanisms underlying this lack of activity have yet been reported for *KRAS^Q61X^*-driven lung cancer, reports from other cancers driven by *RAS^Q61X^* mutations have offered insights and potential avenues for investigation. For example, Marasco et al. showed that *RAS^G12X^* cell lines exposed to RMC-7977 exhibited inhibition of the PI3K-AKT-mTOR pathway; however, *RAS^Q61X^* cell lines did not show such inhibition [109]. Additionally, reactivation or sustained activation of the PI3K-AKT-mTOR pathway has been a mechanism of resistance to other RAS-targeting agents, such as OFF-state inhibitors [60,110]. Sustained or increased PI3K-AKT-mTOR pathway activation upon ON-state RAS targeting may be responsible for treatment resistance in KRAS^Q61X^ lung cancers, given that other reports have suggested that *KRAS^Q61X^* mutations, especially *KRAS^Q61H^*, confer reduced preference for PI3K binding. This suggests that additional therapeutic pressure is needed alongside RMC-7977 to fully shut down RAS signalling in *KRAS^Q61X^*-driven cancers. Finally, other reports have identified that certain *KRAS^Q61X^* mutations alter the conformational dynamics of the switch II region, adopting conformations that restrict access to the switch II pocket, thereby reducing the affinity of RAS inhibitors [111,112].

### KRAS-mutant allele-specific vulnerability to dual ON/OFF-state inhibitors

BBO-11818 binds the switch II/helix 3 pocket of both KRAS (ON) and (OFF). Stahlhut et al. demonstrated that BBO-11818 inhibits MAPK signalling and cell proliferation in both *KRAS*-mutant cancer-derived cell lines and isogenic cell panels, exhibiting broad activity against most KRAS oncoproteins relevant to human cancers [92]. However, its effects were limited in *KRAS^G12R^*- and *KRAS^Q61X^*-mutant models. No significant activity against NRAS and HRAS was observed. A particularly interesting *in vitro* experiment supporting the preference for dual (ON) and (OFF) pan-KRAS inhibitors showed that epidermal growth factor (EGF) stimulation reduces the potency of both BI-2493 (pan-KRAS (OFF) inhibitor) and BBO-11818 in decreasing pERK in *KRAS^G12D^*- and *KRAS^G12V^*-mutant cells, but the fold shift is much lower for BBO-11818. *In vivo* studies demonstrated anti-tumour activity with twice-daily oral administration of BBO-11818 in *KRAS^G12X^*-mutant NSCLC, CRC, and PDAC xenograft models [92]. The phase Ia/b KONQUER-101 trial (NCT06917079) is currently recruiting patients with *KRAS^G12X^*-mutant solid tumours in multiple locations in the U.S.A. and Australia.

### KRAS-mutant allele-specific vulnerability to KRAS-targeted degraders

The pan-KRAS degrader ACBI3, which binds to GDP-bound KRAS, demonstrated potent degradation of WT KRAS and most clinically relevant KRAS oncoproteins, including KRAS^G12A^, KRAS^G12C^, KRAS^G12D^, KRAS^G12V^, KRAS^G13C^, KRAS^G13D^, and KRAS^Q61H^. However, KRAS^G12R^, KRAS^Q61K^, KRAS^Q61L^, and KRAS^Q61R^ were resistant to degradation. The authors attributed this lack of activity to the complete loss of GTPase capacity in the resistant mutants, implying that responsive mutants must have residual GTPase capacity to some extent [103].

### KRAS-mutant allele-specific durability of response to RAS targeting agents

It is now established that certain *KRAS* mutations respond better to RAS-targeted therapy than others. However, the durability of responses among initially responding mutations has yet to be fully documented. One report recently addressed this by comparing time-to-relapse after KRAS inhibition between NSCLC GEMMs harbouring either *KRAS^G12C^* or *KRAS^G12D^* [113]. Given that these two mutants respond similarly to acute ON- and OFF-state (K)RAS inhibition, Huang et al. reported that tumours harbouring either genotype demonstrated comparable rapid reductions in volume (adagrasib and MRTX1133 were used to treat *KRAS^G12C^* and *KRAS^G12D^* mice, respectively). However, endogenous murine lung tumours expressing KRAS^G12D^ relapsed much more quickly than those expressing KRAS^G12C^ after withdrawal of KRAS inhibitor treatment. They conducted a similar experiment without treatment withdrawal, again showing that *KRAS^G12D^*-driven lung tumours became resistant more quickly and that this was associated with higher Ki67. The quicker relapse of *KRAS^G12D^* tumours may reflect the aggressive nature of this mutation, given its association with greater oncogenic potential in lung cancer [21,60]. Furthermore, MRTX1133 targets both the ON and OFF states of KRAS^G12D^ [87], albeit the ON state with less affinity, while adagrasib solely targets the OFF state of KRAS^G12C^ [87]. This further underscores the aggressive nature of *KRAS^G12D^*, as *KRAS^G12D^*-driven lung tumours relapsed quickly despite broad target coverage. Accordingly, it suggests that KRAS inhibition is more durable in tumours harbouring fewer oncopotent KRAS mutations. Given the breadth of KRAS inhibitors in clinical trials, it is timely to examine the durability of different *KRAS*-mutant tumours under RAS-targeted therapeutic pressure, anticipating which mutations are more associated with relapse and aiming to understand which escape mechanisms they engage.

Collectively, these reports provide strong evidence that *KRAS*-mutant alleles have differential sensitivity to KRAS-targeting agents, depending on the mechanism of action of the agents and biochemical properties of the mutant alleles. They are in agreement that KRAS^G12X^ mutants are more vulnerable to agents targeting the ON state, whereas most KRAS^Q61X^ mutants are relatively resistant to several types of KRAS targeting (both inhibition and degradation), highlighting a need for further drug development tailored to this subgroup.

## KRAS-mutant allele-specific sensitivity to upstream modulation and targeting

The development of KRAS^G12C^ (OFF) inhibitors revealed the dependency of this *KRAS* mutation on RTK-driven GEF activity [83,85,105]. This raised the question as to whether other *KRAS* mutations, which are influenced by GEF modulation, are sensitive to upstream therapeutic intervention (Figure 4).

**Figure 4:**
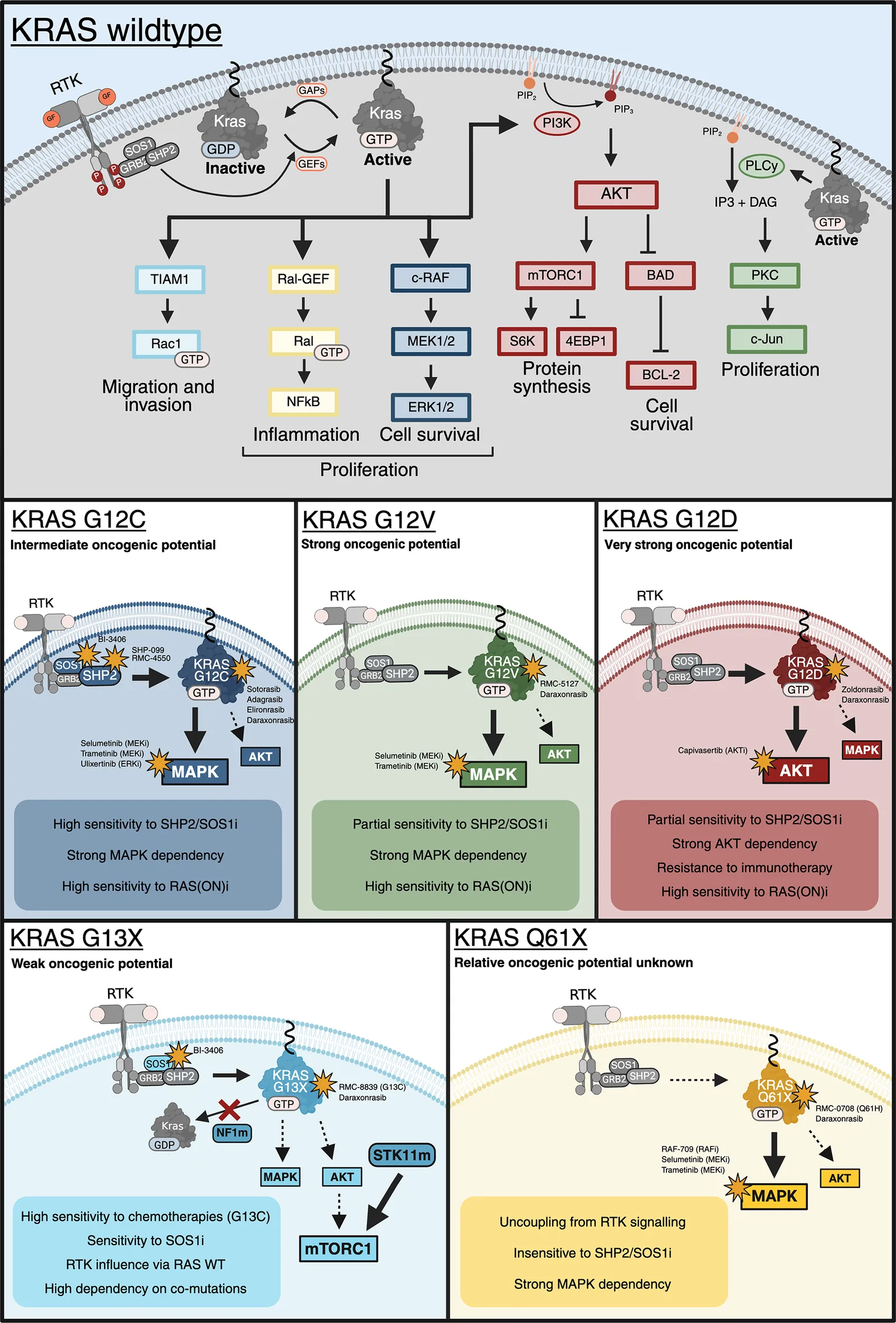
KRAS^WT^ and mutant-specific signalling and targetable vulnerabilities in cancer Upper panel: WT KRAS signalling. Lower panels: mutant-specific signalling, available (K)RAS inhibitors, and unique features and vulnerabilities (inside box). Stars indicate targetable vulnerabilities. Created in BioRender. Tinsley, K. (2027) https://BioRender.com/ffw6ul9. KRAS: Kirsten rat sarcoma; RTK: receptor tyrosine kinase; SOS1: sons-of-sevenless; GRB2: growth factor receptor-bound protein 2; GAP: GTPase-activating protein; GEF: guanine exchange factor; GDP: guanosine diphosphate; GTP: guanosine triphosphate; SHP2: Src homology containing protein tyrosine phosphatase 2; MAPK: mitogen-activated protein kinase; NF1m: neurofibromin gene mutation; STK11m: serine/threonine kinase 11 gene mutation; WT: wild-type.

### SHP2

SHP2 functions upstream of KRAS, promoting or maintaining KRAS-GTP levels through RTK signalling, indicating that targeting this phosphatase confers anti-tumour activity against KRAS mutants that cycle between ON and OFF states. This has been exemplified through studies in which SHP2 inhibitors disable oncogenic signalling by KRAS^G12X^ mutants, most notably KRAS^G12C^. Using an isogenic *RAS*-less MEF model reconstituted with different *KRAS* mutations, Mainardi et al. showed that all *KRAS^G12X^* MEFs and *KRAS^G13D^* MEFs responded to allosteric SHP2 inhibitor SHP099 [114]. However, *KRAS^Q61R^* MEFs remained resistant. This correlated with reductions in pERK1/2 across all *KRAS*-mutant MEFs, except for *KRAS^Q61R^* MEFs. Extending this work, Fedele et al. provided a mechanistic rationale for the difference in SHP2 inhibitor activity between KRAS mutants, showing that *KRAS* mutations with intact GTPase activity, such as *KRAS^G12C^*, were susceptible to SHP099, whereas those with abolished GTPase activity (*KRAS^Q61R^*) were resistant [115]. Interestingly, using an alternative allosteric SHP2 inhibitor, RMC-4550, in genetically complex cancer models, Nichols et al. demonstrated that cancer cell lines harbouring *KRAS^G12C^* and *KRAS^G12A^* mutations, including NSCLC lines, showed exquisite dependence on SHP2 to maintain viability. Cell lines harbouring other KRAS mutations, such as *KRAS^G12D^* and *KRAS^G12V^*, exhibited heterogeneous responses. *KRAS^G13X^*- and *KRAS^Q61X^*-mutant cell lines were markedly resistant to RMC-4550 [116]. In another study that used cell lines from multiple cancer types, including NSCLC, cell lines harbouring *KRAS^G12X^* mutations demonstrated strong reductions in both pERK1/2 levels and viability in response to SHP099, whereas those with *KRAS^G13X^* and *KRAS^Q61X^* mutations did not [117]. The disparity in the response to SHP2 inhibition between *KRAS^G13D^* MEFs and cancer cell lines may be due to cell-type differences. A study on the relationship between *KRAS^Q61H^* and RTK-driven GEF activity reported that this mutation decouples KRAS from RTK regulation. Upon SHP2 inhibition, this unique feature of *KRAS^Q61H^* maintained MAPK pathway activation, rendering cells resistant [45].

Further reports have shown that SHP2 inhibitors combine effectively with KRAS inhibitors, particularly KRAS^G12C^ OFF-state inhibitors. Fedele et al. described how inhibition of SHP2 with SHP099 increases the pool of KRAS^G12C^-GDP, thereby improving target coverage with the KRAS^G12C^ inhibitor ARS-1620, highlighting an effective mutant allele-tailored combination strategy [115]. Additionally, Anastasiou et al. described the combination efficacy of RMC-4550 and the KRAS^G12C^ ON-state inhibitor RMC-4998 (RAS^G12C^-selective ON-state inhibitor) [118]. Both studies also demonstrated strong preclinical evidence that this combination remodelled the tumour microenvironment to enhance PD-1 blockade.

### SOS1

The development of BI-3406, a SOS1 inhibitor, revealed the dependence of *KRAS*-mutant cells on SOS1. Hofmann et al. exposed a large panel of cell lines representing different cancer types to BI-3406 and noted that *KRAS^G12X^* and *KRAS^G13D^*-mutant cell lines were selectively vulnerable [119]. In keeping with SHP2 inhibitor sensitivity profiles, *RAS^Q61X^* mutations conferred resistance to SOS1 inhibition. Using an isogenic model derived from *KRAS^G12C^*-driven H23 cells, an NSCLC cell line, in which *KRAS^G12C^* was replaced with other *KRAS* mutations, the authors confirmed the selective sensitivity of most *KRAS^G12X^* (most notably *KRAS^G12C^* and *KRAS^G12D^*) and *KRAS^G13X^* mutations to BI-3406. *KRAS^Q61H^* and *KRAS^G12R^* mutations, in the presence of this inhibitor, were still able to maintain MAPK signalling and viability. SOS1 inhibition has been reported to improve responses to KRAS^G12C^ inhibition in NSCLC preclinical models. Daley et al. used BI-3406 to prevent adaptive resistance to KRAS^G12C^ inhibition, mediated by RTK and ERK activation [120]. This compound further sensitised drug-tolerant persister cells to KRAS^G12C^ inhibitors, preventing the development of acquired resistance.

Overall, studies of mutant allele-specific sensitivity to upstream signalling inhibition are broadly concordant, with KRAS^G12C^ consistently showing strong dependence on both SOS1 and SHP2 and KRAS^Q61H^ consistently showing independence. These data also provide important insight into KRAS^G13D^ biology and its differential reliance on SOS1 versus SHP2. Given its dependence on GEF activity, KRAS^G13D^ may require robust SOS1 engagement and is therefore potentially recruited and activated through inputs beyond SHP2-dependent signalling. This broader upstream requirement may explain why SHP2 inhibition alone is insufficient in this context.

## KRAS-mutant allele-specific sensitivity to downstream targeting

Several studies examining the complexity of *KRAS*-mutant allele-specific downstream signalling have uncovered that different mutations can signal predominantly through distinct arms of KRAS signalling, with most work focusing on the MAPK and PI3K-AKT-mTOR signalling arms. Overall, these studies suggest that different *KRAS* mutations can be selectively targeted by inhibiting these pathways (Figure 4), highlighting the potential of such inhibition as part of a rational combination approach with KRAS inhibitors.

### MAPK signalling

Several studies have investigated *KRAS*-mutant allele-specific MAPK signalling to determine if certain alleles preferentially signal through the MAPK arm of KRAS signalling and are therefore selectively vulnerable to MAPK blockade. Li et al. analysed NSCLC patient tumours by immunohistochemical staining for pERK1/2 and pMEK1/2 and observed higher MAPK activation in *KRAS^G12C^* (and *KRAS^G12V^*) patients compared with *KRAS^G12D^* patients [121]. They confirmed in both isogenic cell lines and GEMMs of NSCLC that *KRAS^G12C^* models were more vulnerable to MEK1/2 inhibition than *KRAS^G12D^* models. In a different study by Ricciuti et al., despite not observing higher MAPK activation in *KRAS^G12C^* than in *KRAS^G12D^* MEFs, *KRAS^G12C^* MEFs were more sensitive to MEK1/2 inhibition than *KRAS^G12D^* MEFs [21]. In agreement, Ambrogio et al. also reported the same mutation-specific responses in a *KRAS*-mutant MEF panel [122]. Using physiologically relevant models, our group also noted greater sensitivity of human *KRAS^G12C^* NSCLC models to inhibition of ERK1/2 and MEK1/2, while observing comparable MAPK activation downstream of both KRAS^G12C^ and KRAS^G12D^ mutants [60]. Unexpectedly, given the substantial preclinical evidence, Ihle et al. reported that both *KRAS^G12C^* and *KRAS^G12V^* NSCLC patients had poorer survival outcomes than patients harbouring other *KRAS* mutations in response to the RAF inhibitor sorafenib [56]. However, Jänne et al. reported that the addition of the MEK inhibitor selumetinib to a docetaxel regimen greatly increased OS in patients with *KRAS^G12C^* or *KRAS^G12V^* NSCLC, while minimal improvement in OS was observed in patients harbouring other *KRAS* mutations [123].

Alongside its autonomy from upstream signalling nodes, *KRAS^Q61H^* has been reported to alter effector selectivity, with downstream signalling largely mediated by the MAPK cascade. Zhou et al. argued that the structural reason for this is that this amino acid substitution enhances interactions with RAF but decreases interactions with the p110γ catalytic subunit of PI3K [124]. To support this, they reported a comparative analysis of pAKT and pERK expression as readouts of MAPK and PI3K-AKT-mTOR signalling cascades by Western blot and immunohistochemistry in *KRAS^WT^*-, *KRAS^G12D^*-, and *KRAS^Q61H^*-mutant NSCLC tissue specimens, also observing increased activity of RAF and MEK inhibitors in this mutant subtype. This preferential engagement with the MAPK pathway, coupled with abolished GTPase activity and increased nucleotide exchange, may confer oncogene-induced toxicity given that a previous report demonstrated that models driven by *KRAS^Q61H^* are less proliferative compared with those driven by other mutant alleles [21]. This may explain why *KRAS^Q61H^* mutations are rare in NSCLC. Addressing this speculation requires the use of GEMMs or inducible models of *KRAS^Q61H^* to assess its immediate impact since advanced cancer cell lines evolve and fine-tune signalling to a ‘sweet spot’ to support malignant progression.

Co-mutational context has also been shown to influence the sensitivity of *KRAS*-mutant NSCLC to MAPK blockade. Loss of *STK11* has been reported to increase the sensitivity of preclinical *KRAS*-mutant models to ERK inhibition. In isogenic *STK11*-knockout models, *STK11* loss enhanced the cytotoxic effect of the ERK inhibitor SCH772984 by enabling suppression of p90 S6K phosphorylation. Although this effect was demonstrated in *KRAS^G12C^* models with *STK11* loss, activity was also observed in models derived from a *KRAS^G12D^* GEMM on an *STK11*-knockout background [125]. This is notable because *KRAS^G12D^* NSCLC cell lines have shown reduced sensitivity to MAPK inhibition compared with those driven by *KRAS^G12C^* [60]. Given that *STK11* alterations occur in *KRAS^G12D^* NSCLC, albeit at lower frequency than in other mutant alleles (Figure 3), these findings raise the question of whether this molecular subgroup may derive clinically meaningful benefit from MAPK pathway inhibition. In agreement, a separate study using a physiologically relevant panel of *KRAS*-mutant NSCLC cell lines with endogenous *STK11* alterations showed that *STK11* loss increased sensitivity to MAPK inhibition with trametinib. In these models, MAPK inhibition hyperactivated JNK-mediated stress signalling, thereby creating a therapeutically induced dependency on MCL-1. Combined inhibition of the MAPK pathway and MCL-1 produced strong anti-tumour activity in *STK11*-mutant cell lines, irrespective of *KRAS*-mutant allele [126]. By contrast, *KEAP1* loss increased resistance to MAPK inhibition with trametinib in *KRAS*-mutant NSCLC cell lines by attenuating drug-induced reactive oxygen species and enabling cell survival despite MAPK pathway suppression through altered glutathione biosynthesis [127].

Finally, combining KRAS^G12C^ OFF-state inhibitors with MAPK pathway inhibitors has demonstrated strong anti-tumour responses in preclinical models [21]. Given that *KRAS^G12C^* is already selectively vulnerable to MAPK inhibition and OFF-state inhibitors, this type of combination is a good example of tailoring a combination strategy to the specific KRAS mutation subtype.

### PI3K-AKT-mTOR signalling

Preclinical investigation into the PI3K-AKT-mTOR arm of KRAS signalling has uncovered a link between this pathway and the higher oncogenic potential of *KRAS^G12D^*. Our group recently used a series of cell lines and GEMMs to compare oncogenicity and downstream pathway activation in *KRAS^G12D^*- versus *KRAS^G12C^*-mutant NSCLC [60]. It was shown that *KRAS^G12D^* is a more ‘oncopotent’ variant at the stage of tumour initiation. A relevant association with this phenotype was that *KRAS^G12D^* initiation models exhibited significant activation of the PI3K-AKT-mTOR pathway. However, at later stages of tumourigenesis, this feature was equivalent *in vitro* and *in vivo* between models harbouring each allele. Nonetheless, advanced *KRAS^G12D^* NSCLC models remained vulnerable to AKT inhibition, with synergy observed when combining mutant-selective KRAS inhibition and AKT inhibition. In agreement, Ihle et al. demonstrated increased activation of the PI3K-AKT-mTOR pathway in an isogenic panel of cell lines expressing KRAS^G12D^, with KRAS^G12C^ as a comparator [56]. Using molecular modelling studies of the *KRAS*-mutant proteins, they provided a rationale for the increased engagement of KRAS^G12D^ with the PI3K-AKT-mTOR pathway. They explained that, upon binding to PI3K, the switch II regions of both KRAS^WT^ and KRAS^G12C^ adopt a conformation that renders GTP susceptible to hydrolysis, thereby reducing KRAS activation. However, the large, bulky chain associated with KRAS^G12D^ forms a hydrogen bond with PI3K in the switch II region, preventing GTP hydrolysis and maintaining the ON state of KRAS^G12D^ and its signalling through PI3K.

Given that PI3K-AKT-mTOR signalling is a critical pathway driving tumour formation in *KRAS*-mutant NSCLC [128,129], the molecular modelling data from Ihle et al. raise an interesting question about how other KRAS-mutant proteins, such as KRAS^G12C^, which are susceptible to hydrolysis upon engagement with PI3K, can promote lung tumourigenesis. In pancreatic cancer, KRAS^G12R^ can still promote tumour formation, despite being unable to bind PI3K and activate the PI3K-AKT-mTOR signalling pathway [130]. Patient genomics data analyses have revealed that *KRAS^G12R^*-driven PDAC tumours are enriched for mutations in this pathway, effectively compensating for the impaired KRAS^G12R^–PI3K interaction [131]. In NSCLC, several studies have highlighted that certain *KRAS* mutations, most notably those with weaker oncogenicity, such as *KRAS^G12C^, KRAS^G13C^*, and *KRAS^G13D^*, are associated with mutations in *STK11* and *KEAP1* [21,132]. Mutations in these proteins promote mTOR pathway activation since they are negative regulators of mTOR [133,134]. Our group confirmed the importance of *STK11* mutations in maintaining *KRAS^G13X^*-driven tumour growth by restoring *STK11* WT function in an *STK11*-mutant cell line. We observed reduced mTOR signalling, which coincided with impaired spheroid expansion, underlining the dependence of *KRAS^G13X^* mutations on co-mutations [44].

In summary, KRAS-mutant alleles can selectively signal through distinct arms of KRAS signalling, creating targetable dependencies that can be therapeutically exploited as part of combination therapies.

## Other therapeutic modalities

While most research has focused on targetable vulnerabilities in the KRAS pathway, there have been reports of *KRAS*-mutant allele-specific vulnerabilities to standard-of-care treatments (Figure 4).

### Chemotherapy

The literature indicates that most *KRAS* mutations are not strongly predictive of sensitivity to classic chemotherapy. Instead, as already explained, they are much more predictive of resistance to targeted therapies (such as KRAS inhibitors). However, there are emerging (but still limited) data on *KRAS*-mutant allele-specific chemosensitivity. In a small study by Garassino et al., a KRAS^WT^ NSCLC cell line was modified to express one of KRAS^G12C^, KRAS^G12D^, or KRAS^G12V^. All cell lines displayed comparable growth kinetics; however, they differed in their responses to chemotherapeutics. In relation to H1299-WT, H1299-G12C was associated with resistance to cisplatin and increased sensitivity to taxol and pemetrexed, while H1299-G12D and H1299-G12V were associated with increased resistance to taxol and increased sensitivity to cisplatin, respectively [135]. To understand the mechanism behind KRASG12C-specific resistance to cisplatin, Caiola et al. reported that H1299-G12C cells showed less γH2AX (a marker of DNA damage) upon cisplatin treatment [136]. They found that KRAS^G12C^ can stimulate base excision repair to rapidly remove platinum from DNA, even before DNA damage occurs. Co-treating H1299-G12C cells with cisplatin and methoxyamine (an inhibitor of base excision repair) restored sensitivity to cisplatin. In a clinical report, no difference in survival was seen in patients with *KRAS^G12C^* NSCLC receiving first-line platinum-based chemotherapy (PBC) compared with patients affected by other *KRAS* mutations [137]; however, combining PBC with immunotherapy increased OS in patients with *KRAS^G12C^* NSCLC compared with *KRAS^non-G12C^* and *KRAS^WT^* [138]. This discrepancy between *in vitro* and clinical data, which was also apparent when translating MAPK inhibition to the clinic as a monotherapy for *KRAS^G12C^* and *KRAS^G12V^* patients, highlights a limitation of *in vitro* monoculture models [21,56,60,121–123]. Rather, using co-culture models or immunocompetent mouse models that faithfully recapitulate the tumour microenvironment would provide more meaningful insight into certain tumour vulnerabilities.

Our group recently showed, through a compound library screen, that *KRAS^G13C^* confers broad chemosensitivity in NSCLC cell lines compared with other *KRAS*-mutant genotypes. We confirmed this vulnerability in isogenic models and in a retrospective analysis of survival outcomes of patients who received only first-line chemotherapy [44]. We propose that this enhanced sensitivity is due to the weaker oncogenic properties of *KRAS^G13C^* and speculate that this mutation cannot counteract the cytotoxic effects of chemotherapy as effectively as more oncogenic *KRAS* mutations, possibly through increased apoptotic resistance. We chose docetaxel as a model chemotherapy agent and combined it with the KRAS^G13C^-selective RAS (ON) inhibitor RMC-8839. Strong co-operation was observed between the two compounds, leading to robust tumour regression *in vivo* [44].

### Immunotherapy

With regard to immune checkpoint blockade, evidence suggests that *KRAS^G12D^*-mutant NSCLC exhibits a resistant phenotype compared with other *KRAS* mutational subtypes. Firstly, in a major clinical study comparing *KRAS^G12D^* NSCLC patients with *KRAS^non-G12D^* NSCLC patients, survival with PD-(L)1-based therapies was worse in *KRAS^G12D^* patients. Consistent with this inferior response, downstream analyses of patient samples showed reduced PD-L1 IHC staining and lower TMB in *KRAS^G12D^* patients, both of which are biomarkers of immunotherapy response [49]. Laboratory investigations suggest that a *KRAS^G12D^* mutation creates a more immunosuppressive environment in NSCLC than other *KRAS* mutations. Firstly, Huang et al. used *KRAS^G12C^* and *KRAS^G12D^* GEMMs to compare the impact of these mutations on the TME [113]. They performed gene expression analysis and observed higher expression of genes involved in lymphocyte chemotaxis, CD8^+^ T-cell function, antigen presentation, and antibody production in *KRAS^G12C^*-driven tumours. Using flow cytometry, they characterised immune infiltration in lung tissues and saw higher CD3^+^ and CD8^+^ T cells, higher expression of IFN-γ by CD8^+^ T cells, and higher MHC-I and PD-L1 expression on tumour cells in the lung of *KRAS^G12C^* mice, rendering lung tumours with this *KRAS* mutation more immunogenic. Furthermore, they observed increased expression of Ly6G+ myeloid cells in the lungs of *KRAS^G12D^* mice, which are known to promote immunosuppression. Liu et al. also conducted their own analysis using isogenic NSCLC models and reported that a *KRAS^G12D^* mutation negatively correlated with PD-L1 and CXCL10/CXCL11 chemokine secretion [139]. This led to reduced CD8^+^ T-cell infiltration due to KRAS^G12D^-driven activation of the PI3K-AKT-mTOR pathway and down-regulation of high mobility group protein A2 (HMGA2). They concluded that paclitaxel improved PD-L1-based therapy response by up-regulating HMGA2 expression, thereby increasing CXCL10/CXCL11 secretion and CD8^+^ T-cell infiltration in a *KRAS^G12D^* NSCLC mouse model.

In contrast with *KRAS^G12D^*, other *KRAS* mutational subtypes derive meaningful clinical benefit from immune checkpoint blockade. Despite initial concerns about intolerable hepatotoxicity with sotorasib–immunotherapy combinations, in *KRAS^G12C^*-mutant NSCLC patients, adagrasib–pembrolizumab and olomorasib–pembrolizumab combinations have proven tolerable and demonstrated encouraging activity in the KRYSTAL-7 and LOXO-RAS-20001 phase II studies, both showing progression-free survival of approximately 11 months [70,74]. In a real-world study of *KRAS^G12V^*-mutant NSCLC, John et al. reported substantial differences in outcomes between patients who received chemotherapy alone and those who received immune checkpoint inhibitors alone or in combination with chemotherapy, concluding that this population derives substantial clinical benefit from immune checkpoint blockade [51].

Although *KRAS*-mutant alleles appear to confer differential sensitivity to immunotherapy, co-mutational context can substantially modify these responses. The role of *TP53* mutations remains incompletely resolved. Some studies reported no clear association between *TP53* status and response to immune checkpoint blockade in *KRAS*-mutant NSCLC [140,141]. In contrast, other studies have associated *TP53* co-mutation with improved survival compared with *TP53^WT^* disease, reflected by higher tumour mutational burden, increased PD-L1 expression, and greater CD8^+^ T-cell infiltration [142,143]. This conflicting evidence may reflect heterogeneity within the *TP53* mutational landscape and indicate that *TP53* mutations are not reliable predictive biomarkers of immunotherapy response. Conversely, most clinical reports agree regarding the negative impact of *STK11* and *KEAP1* mutations and their reliability as biomarkers. These are generally associated with impaired immunotherapy responsiveness, consistent with an immunosuppressive tumour microenvironment characterised by increased myeloid-cell infiltration and reduced CD8^+^ T-cell infiltration and PD-L1 [140,141,144,145].

In summary, beyond therapeutic targeting of individual KRAS signalling nodes, *KRAS* mutations can also shape sensitivity to non-KRAS-directed treatments. This has important clinical implications, particularly as emerging preclinical evidence supports combining KRAS inhibitors with frontline treatment regimens [146,147].

## Concluding remarks

There is compelling evidence to support the idea that *KRAS*-mutant alleles should be treated as separate entities. Individual *KRAS* mutations have specific vulnerabilities: at the level of KRAS itself; arising from maintained upstream interactions or their absence; and through selected downstream signalling (Figure 4). For example, *KRAS^G12C^* confers sensitivity to several targetable nodes of KRAS signalling, such as upstream targeting of SHP2 and SOS1, both OFF- and ON-state KRAS inhibition, and downstream MAPK inhibition (Figure 4). Accordingly, certain mutations are associated with distinct resistance profiles. Notably, *KRAS^G12D^* and *KRAS^Q61H^* confer resistance to immunotherapy and upstream targeting, respectively (Figure 4). Several of these mutation-specific sensitivities seem to be governed by the biochemical features of each individual *KRAS* mutation (Figure 2): the level of residual GTPase activity and nucleotide exchange and the binding affinity to effector proteins. Rational combination therapies with KRAS inhibitors should be contextualised to these mutation-specific features to maximise patient treatment outcomes.

At the time of writing, reports of *KRAS* mutation-specific vulnerabilities largely focus on the MAPK and PI3K-AKT-mTOR pathways. Given that KRAS activates several downstream pathways and that multiple *KRAS* mutations are regulated by RTKs, there is a knowledge gap regarding whether specific mutations are vulnerable to the inhibition of either distinct RTKs or other downstream signalling pathways. In support of this statement, Ihle et al. have shown that KRAS^G12C^ can activate RAL signalling more strongly than KRAS^G12D^ in isogenic lung models. Additionally, in CRC, KRAS^G13D^ remains partially susceptible to EGFR antibody-based therapy.

Another understudied area is how *KRAS* mutations can differentially promote or inhibit cell-intrinsic or -extrinsic processes. With strong evidence supporting the immunosuppressive functions of *KRAS^G12D^*, other hallmarks of cancer should be explored, including apoptotic resistance, angiogenesis, and metastasis. Understanding how KRAS mutations can promote these processes from a drug-targeting perspective can also inform the development of effective combination therapeutic regimens. For example, Hung et al. showed in a lung cancer isogenic model that KRAS^G12V^ promoted increased migration compared with KRAS^G12D^, which was linked to higher Wnt signalling [148]. A retrospective clinical study by Urtecho et al. described how NSCLC metastatic lesions in the liver, bone, and brain had a higher incidence of *KRAS^Q61X^* mutations [149].

To conclude, while we have already learned detailed information about therapeutically targetable susceptibilities that arise from distinct *KRAS* mutations, we are still not close to a complete picture. More research efforts in the areas suggested in the present review should inform rational, allele-specific therapeutic opportunities involving KRAS inhibitors, spanning different cancer histologies from prevention through to advanced disease.

## Open Access

Open access for this article was enabled by the participation of the University of Manchester in an all-inclusive Read & Publish agreement with Portland Press and the Biochemical Society under an agreement with JISC.

## Abbreviations

AAV: adeno-associated virus
CRC: colorectal cancer
EGF: epidermal growth factor
EGFR: epidermal growth factor receptor
GAPs: GTPase-activating proteins
GEMMs: genetically engineered mouse models
GEFs: guanine nucleotide exchange factors
GTPase: guanosine triphosphatase
HRAS: Harvey-Ras
HVR: hypervariable region
KRAS: Kirsten-Ras
LOF: loss-of-function
LUAD: lung adenocarcinoma
MEFs: mouse embryonic fibroblasts
NRAS: Neuroblastoma-Ras
NSCLC: non-small cell lung cancer
OS: overall survival
PBC: platinum-based chemotherapy
P-loop: phosphate-binding loop
RTKs: receptor tyrosine kinases
SHP2: SRC homology-containing protein tyrosine phosphatase 2
VHL: von Hippel-Lindau
WT: wild-type
